# Genetic Characterization of Carbapenem-Resistant *Acinetobacter* spp. Isolated from Diseased Companion Animals in Japan

**DOI:** 10.3390/antibiotics15040329

**Published:** 2026-03-24

**Authors:** Saki Harada, Mari Matsuda, Yuta Hosoi, Taimu Toyama, Michiko Kawanishi, Hideto Sekiguchi

**Affiliations:** National Veterinary Assay Laboratory, Ministry of Agriculture Forestry and Fisheries, 2-1-22 Kannondai, Tsukuba 305-8535, Japan

**Keywords:** *Acinetobacter* spp., carbapenemase, WGS, companion animals, JVARM

## Abstract

Background/Objectives: Carbapenem-resistant *Acinetobacter* spp. represent an emerging concern in human medicine; however, their epidemiology and genetic backgrounds in companion animals in Japan remain unclear. This study aimed to determine the prevalence of carbapenem resistance among *Acinetobacter* spp. isolated from diseased dogs and cats and elucidate the underlying genetic mechanisms. Methods: In this surveillance study conducted as part of the Japanese Veterinary Antimicrobial Resistance Monitoring (JVARM) program, 139 isolates were collected from diseased companion animals across Japan (84 from dogs and 55 from cats) during 2020, 2021 and 2023. Antimicrobial susceptibility testing was performed for seven antimicrobials and carbapenem-resistant isolates (meropenem MIC ≥ 8 μg/mL) underwent whole-genome sequencing to identify resistance genes, genomic contexts, and associated mobile genetic elements. Results: Resistance rates to all tested antimicrobials were below 20%. Meropenem resistance was detected in three isolates: one from a dog and two from cats. These resistant strains were identified as *A. radioresistens*, *A. proteolyticus*, and *A. johnsonii*, all harboring carbapenemase genes. The *A. radioresistens* isolate carried chromosomal *bla*_OXA-23_, the *A. proteolyticus* isolate carried *bla*_OXA-58_, and the *A. johnsonii* isolate possessed a plasmid containing *bla*_NDM-1_ and *bla*_OXA-58_. This represents the first report of *bla*_NDM-1_-harboring *Acinetobacter* isolate from companion animals in Japan. Conclusions: Carbapenem-resistant *Acinetobacter* spp. remain rare in companion animals in Japan; however, insertion sequence mobility may promote resistance gene dissemination. As carbapenems are not approved for veterinary use in Japan, strict antimicrobial stewardship and appropriate hygiene management are essential.

## 1. Introduction

*Acinetobacter* spp. are nonmotile, Gram-negative bacteria that include more than 50 species and are obligate aerobes. These bacteria are ubiquitous in nature and are commensals on the skin of healthy humans and animals. However, *Acinetobacter* spp. can also act as opportunistic pathogens in immunocompromised individuals, and are a major cause of nosocomial infections via contaminated medical equipment, including catheters and ventilators [1]. *Acinetobacter* spp. are associated with various types of infections in dogs and cats such as pyoderma, necrotizing fasciitis and urinary tract infection [2].

Carbapenems are broad-spectrum β-lactam antibacterial agents considered the last-resort treatment for infections caused by multidrug-resistant Gram-negative bacteria, including *Acinetobacter* spp. [3]. The World Health Organization (WHO) released the updated Medically Important Antimicrobials (MIA) list as a One Health risk-management tool, underscoring prudent use and identifying drug classes authorized only for human use [4]. In Japan, carbapenems are not approved for veterinary use, but those approved for human use are occasionally administered to companion animals based on veterinary decision [5]. Although the carbapenem resistance rate of *Acinetobacter* spp. in humans in Japan remains low [6], the global emergence of carbapenem-resistant *Acinetobacter* spp. in recent years has led to an increasing number of difficult-to-treat cases [7].

Mechanisms of carbapenem resistance include hydrolysis of antimicrobials by bacterial enzymes (carbapenemases), reduced antimicrobial influx due to altered or lost porins in the outer membrane, and increased activity of efflux pumps [8]. Ambler class D carbapenemases such as OXA-23 and OXA-58 are frequently detected in human isolates of *A. baumannii*, and NDM-1 and IMP-1, Amber class B metallo-beta-lactamases, have also been reported in *Acinetobacter* spp. including *A. baumannii* [9,10]. OXA-23 and OXA-58 have also been detected in *Acinetobacter* spp. from dogs and cats and IMP-1 has been detected in dogs and cats in Japan [11,12,13]. In addition, *Escherichia coli* from dogs carrying the *bla*_NDM-5_ gene have been reported in Japan [14]. These genes are frequently located on plasmids, raising concerns about horizontal transfer.

Although information on the occurrence and antimicrobial resistance of *Acinetobacter* spp. in companion animals remains limited, an increasing number of studies—particularly from Europe—have reported their isolation from dogs and cats. Carbapenemase-producing strains carrying *bla*_OXA23_ or *bla*_OXA58_ have been identified in companion animals in France and Germany, raising concerns regarding their potential role as reservoirs for antimicrobial resistance within the One Health interface [15]. Together, these findings highlight the need for continued surveillance and molecular characterization of *Acinetobacter* spp. in companion animal populations.

Considering the close relationship between companion animals and humans, and the critical role that companion animal surveillance plays in One Health efforts to combat antimicrobial resistance, the Japanese Veterinary Antimicrobial Resistance Monitoring (JVARM) has collected *Acinetobacter* spp. from diseased dogs and cats and investigated their antimicrobial susceptibility. In this study, we assessed the carbapenem susceptibility of *Acinetobacter* spp. in Japanese companion animals and performed genomic characterization of carbapenem-resistant strains using whole-genome sequencing.

## 2. Results

### 2.1. Bacterial Isolation

In 2020, 2021 and 2023, a total of 139 *Acinetobacter* spp. were isolated, including 84 from dogs and 55 from cats; 20 *Acinetobacter* species were identified, with *A. baumannii* being the most prevalent (dogs: 22.6%, cats: 29.1%), followed by *A. pittii* (dogs: 21.4%, cats: 20.0%), *A. radioresistens* (dogs: 14.3%, cats: 7.3%), *A. johnsonii* (dogs: 6.0%, cats: 9.1%), *A. ursingii* (dogs: 4.8%, cats: 7.3%) and *A. lwoffii* (dogs: 7.1%, cats: 3.6%) (Table 1). In dogs, 44 strains (52.4%) were isolated from skin and 40 (47.6%) from urine, whereas in cats, 15 strains (27.3%) were isolated from skin and 40 (72.7%) from urine. Although the JVARM system aimed to collect *Acinetobacter* spp. evenly from clinics nationwide, the final distribution of isolates was skewed toward submissions from urban regions.

### 2.2. Phenotypic Characterization of Acinetobacter spp. Isolated from Dogs and Cats in Japan

The number of isolates resistant to each antimicrobial agent is shown in Table 2, with resistance rates ranging from 0% to 36.4% depending on the agent. Resistance rates were significantly higher in cats than in dogs for gentamicin (dogs 7.1% vs. cats 21.6%), tetracycline (dogs 7.1% vs. cats 23.6%), ciprofloxacin (dogs 15.5% vs. cats 36.4%), and sulfamethoxazole/trimethoprim (dogs 15.5% vs. cats 32.7%). Among the 139 isolates, 22 isolates exhibited resistance to three or more antimicrobial classes. The MIC distributions for all tested agents are provided in Supplementary Table S1.

One meropenem-resistant strain (*A. radioresistens*) was detected in dogs and two strains (*A. proteolyticus* and *A. johnsonii*) in cats (Table 3). All meropenem-resistant strains were isolated from urine. The resistance phenotypes of these three strains differed: the dog-derived strain AC-1 was resistant to gentamicin (MIC: >16 μg/mL) and the cat-derived strain AC-2 was resistant to ciprofloxacin (MIC: >8 μg/mL). The cat-derived strain AC-3 exhibited a multidrug resistance phenotype, with resistance to cefotaxime (MIC: >64 μg/mL), gentamicin (MIC: 64 μg/mL), tetracycline (MIC: 32 μg/mL) and ciprofloxacin (MIC: 4 μg/mL).

In addition, resistance rates were compared between isolates obtained from urine and skin samples within each host species. As summarized in Supplementary Table S2, no statistically significant differences were observed between urine- and skin-derived isolates for any antimicrobial agent in dogs or cats. In dogs, urine isolates showed higher resistance proportions to cefotaxime, gentamicin, and ciprofloxacin, although these differences did not reach statistical significance. In cats, interpretation was limited by the small number of skin isolates (*n* = 15), and no clear differences were detected.

### 2.3. Antimicrobial Resistance Genes and Virulence Factors

Genomic analysis of meropenem-resistant strains revealed that AC-1 carried *bla*_OXA-23_, AC-2 carried *bla*_OXA-58_ and *bla*_OXA-286-like_ (*bla*_OXA-674_), and AC-3 carried *bla*_NDM-1_, *bla*_OXA-58_ and *bla*_OXA-211-like_ (*bla*_OXA-651_). According to the Beta-Lactamase Database (BLDB) [16], *bla*_OXA-23_, *bla*_OXA-211-like_ and *bla*_OXA-674_ are the intrinsic resistance genes in *A. radioresistens*, *A. johnsonii* and the corresponding *Acinetobacter* species, respectively.

In all these strains, multiple virulence-associated genes were commonly detected, including several *pil* genes (*pilB*, *pilG*, *pilN*, *pilT*, *pilV*, *pilW*) related to type IV pili, *lpx* genes (*lpxA*, *lpxB*, *lpxC*, *lpxD*, *lpxK*, *lpxO*) related in lipopolysaccharide (LPS) biosynthesis, and *bfmRS* associated with biofilm regulation. Additionally, strains AC-1 and AC-3 carried *ompA*, a gene involved in adherence to and invasion of epithelial cells.

### 2.4. Sequencing and Assembly Quality

A summary of sequencing depth, read quality, and assembly metrics for the three meropenem-resistant isolates is provided in Supplementary Tables S3–S5. Illumina and Nanopore read sets exhibited high read quality and sufficient depth for hybrid assembly, with total short-read bases ranging from 327 to 929 Mb and long-read bases from 49 to 488 Mb. Hybrid assemblies showed N50 values of 2.5–3.3 Mb, L50 = 1, and high completeness (99.2–100%) with low contamination (<1%) based on QUAST, BUSCO and CheckM, consistent with high-quality assemblies. These metrics confirm that the assemblies used for genomic analyses were of high accuracy and completeness.

### 2.5. Genomic and Plasmid Characterization of Strain AC-1

**Figure 1:**
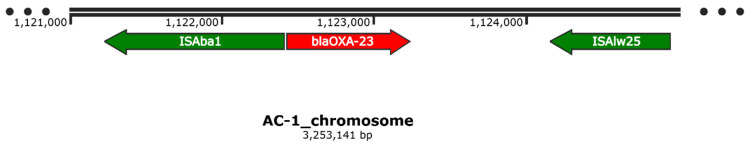
Genetic environment of a portion of the AC-1 chromosome. The *bla*_OXA-23_ gene (red boxes) and insertion sequences (ISs) (green filled boxes) are indicated. Ellipses (“…”) indicate genomic regions not shown (truncated for clarity).

Strain AC-1 was isolated in 2023 from the urine of an 11-year-old dog. AC-1 was found to comprise a chromosome of 3,253,141 bp and eight plasmids ranging from 4237 to 165,234 bp, with antimicrobial resistance genes detected on only one plasmid. The carbapenem resistance gene *bla*_OXA-23_ was located on the chromosome. IS*Aba1* was detected 16 bp upstream of *bla*_OXA-23_ and IS*Alw25* was found 916 bp downstream (Figure 1).

### 2.6. Genomic and Plasmid Characterization of Strain AC-2

**Figure 2:**
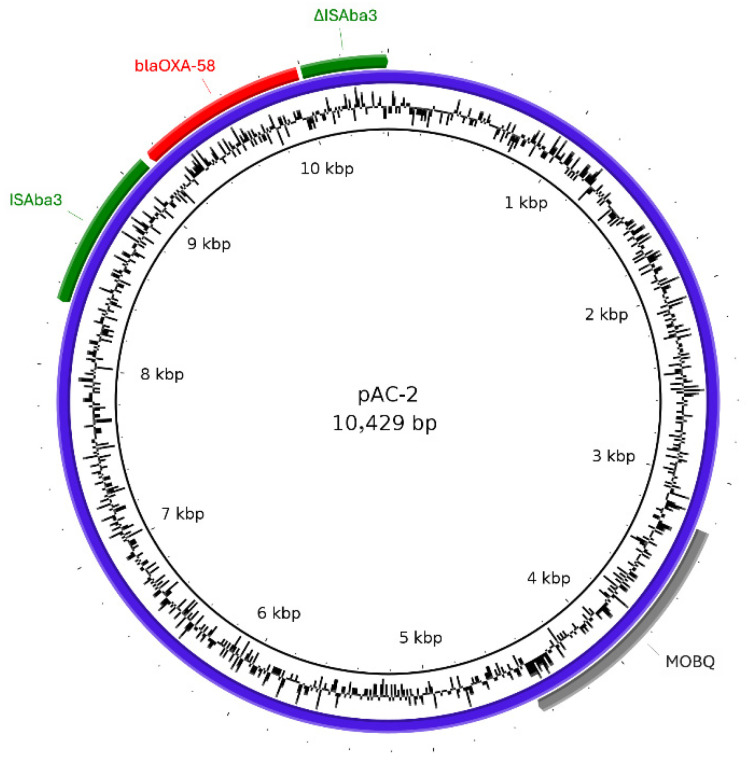
Circular map of the pAC-2 plasmid. The blue ring represents the pAC-2 plasmid sequence. Resistance genes (red boxes), insertion sequences (green boxes), and *MOBQ* gene (gray boxes) are indicated. The inner ring indicates the GC content.

Strain AC-2 was isolated in 2021 from the urine of a 3-year-old cat. The AC-2 genome comprised a 4,580,074 bp circular chromosome and eight plasmids, although only one plasmid was found to carry antimicrobial resistance genes. Plasmid pAC-2, with replicon type R3-T61, was 10,429 bp in size and carried *bla*_OXA-58_. A circular map of pAC-2 is shown in Figure 2. The *bla*_OXA-58_ gene was flanked upstream by a full copy of IS*Aba3* and downstream by a partial copy of IS*Aba3* (427 bp). In this plasmid, the *MOBQ* gene, which encodes a MobQ family relaxase, was detected; however, an *oriT* was not identified.

### 2.7. Genomic and Plasmid Characterization of Strain AC-3

**Figure 3:**
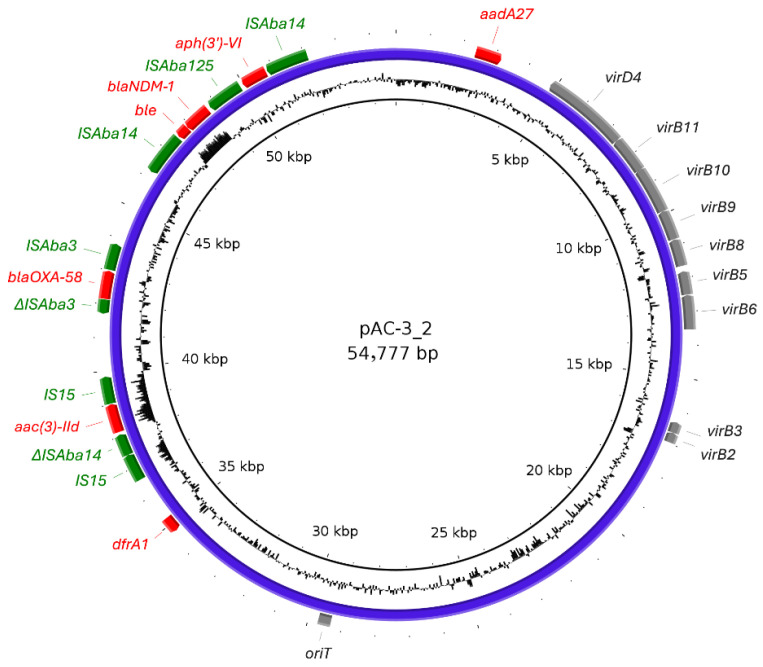
Circular map of the pAC-3_2 plasmid. The blue ring represents the pAC-3_2 plasmid sequence. Resistance genes (red boxes), insertion sequences (green boxes), and the origin of transfer (*oriT*), type IV secretion system (T4SS), and type IV coupling protein (T4CP) gene (*virD4*) (gray filled boxes) are indicated. The inner ring indicates the GC content.

Strain AC-3 was isolated in 2023 from the urine of a 1-year-old cat. Hybrid assembly of short and long reads of the AC-3 strain generated a 3,288,835 bp circular chromosome and four plasmids, with sizes ranging from 4135 to 370,800 bp and GC contents between 37.01% and 42.44%. Among these, three plasmids (pAC-3_1 to pAC-3_3) harbored antimicrobial resistance genes. The replicon type of pAC-3_2 was identified as R3-T20; this plasmid was 54,777 bp in size and had a GC content of 37.17%. The plasmid carried the antimicrobial resistance genes *dfrA1*, *aac(3)-IId*, *bla*_OXA-58_, *bla*_NDM-1_ and ap*h(3′)-VI*. In contrast, the largest plasmid pAC-3_1 (370,800 bp), which had no identifiable replicon type, carried only *tet(H)*. A circular map of pAC-3_2 is shown in Figure 3. In pAC-3_2, *bla*_NDM-1_ was flanked upstream by IS*Aba14* and the *ble* genes, and downstream by IS*Aba125*, the aminoglycoside resistance gene *aph(3′)-VI* and another copy of IS*Aba14*. Both copies of IS*Aba14* contained 50 bp inverted repeat sequences at their ends. Upstream of this *bla*_NDM-1_ containing structure, another region containing *bla*_OXA-58_ was identified, flanked by a partial copy of IS*Aba3* (427 bp) upstream and a complete copy of IS*Aba3* downstream. Additionally, the plasmid carried an origin of transfer (*oriT*), a type IV secretion system (T4SS) region, and a type IV coupling protein (T4CP) gene, *virD4.*

## 3. Discussion

As reported in previous Japanese studies [17], *Acinetobacter* spp. isolated from dogs and cats in Japan showed species diversity. *A. baumannii*, the most clinically relevant species in human nosocomial infections, accounted for approximately 25% of the isolates collected in this study, while non-*A. baumannii Acinetobacter* spp. comprised approximately 75%. In contrast, a study in France [15], although sample collection methods differed, reported that *A. baumannii* accounted for the majority of isolates, suggesting that species distribution may vary by region. Previous reviews have shown that *Acinetobacter* spp. are capable of colonizing both humans and animals and can act as opportunistic pathogens in multiple host species [15]. Given the close contact between companion animals and humans, pets may be exposed to similar environmental sources of *Acinetobacter*, and occasional sharing of bacterial strains or resistance genes is possible. From a One Health perspective, the presence of clinically relevant *Acinetobacter* spp. in companion animals is noteworthy because antimicrobial-resistant strains or resistance determinants could circulate between animals, humans, and their shared environments.

Globally, multidrug-resistant *Acinetobacter* spp., particularly *A. baumannii*, are recognized as major AMR threats and are often associated with high resistance rates in human healthcare settings. In contrast, the resistance rates to all tested agents were less than 20% for all agents in this study, indicating that the susceptibility of *Acinetobacter* spp. derived from dogs and cats in Japan remains generally high. Although the underlying factors cannot be determined from our data, these findings indicate a comparatively low antimicrobial resistance burden in this population. Carbapenem-resistant strains were isolated from 3 of 139 (2.2%) isolates over a 3-year sampling period. In France, 7 of 49 (14.2%) isolates from diseased dogs and cats over a 5-year period were carbapenem-resistant [15]. In Germany, where only *A. baumannii* was collected, 3 of 223 (1.3%) strains derived from dogs and cats were carbapenem-resistant over a 13-year period [18]. Although there are few reports of nationwide surveillance of companion animals-associated *Acinetobacter* spp. and species distribution likely varies from region to region, the isolation rate of carbapenem-resistant *Acinetobacter* spp. in dogs and cats in Japan appears to be relatively low. To interpret these findings appropriately, it is important to consider the structure of the surveillance dataset. Because only one isolate per animal per clinic was included in this analysis, it was not possible to determine whether any of the carbapenem-resistant isolates were associated with nosocomial infections. The three meropenem-resistant isolates originated from three different veterinary clinics. This indicates that the detection of carbapenem-resistant *Acinetobacter* spp. in this study did not result from a single-facility cluster but rather sporadic occurrences across independent clinical settings.

Previous studies [15] from Europe have documented carbapenemase-producing *Acinetobacter* spp. in companion animals. In France, most carbapenem-resistant *A. baumannii* isolates from dogs and cats carried *bla*_OXA-23_, occasionally accompanied by *bla*_OXA-58_. In contrast, investigations from Germany reported a higher proportion of *bla*_OXA-58_-harboring isolates, predominantly associated with ST1 and ST25 lineages. Together, these findings indicate that the predominant carbapenemase types vary across European regions. In contrast to European reports in which carbapenemase-producing *A. baumannii* are commonly detected, no carbapenemase genes were identified in the *A. baumannii* isolates in our study, with all carbapenemase-positive strains instead belonging to non-baumannii species. This difference suggests that the epidemiology of carbapenemases in companion animals in Japan may differ substantially from that in Europe. Because these resistant isolates were non-*A. baumannii*, MLST sequence types could not be determined due to the lack of standardized MLST schemes for these species, and therefore direct comparison with European ST lineages is not applicable.

In Japan, carbapenem antibiotics are not approved for veterinary use; however, in companion animal clinics, carbapenems approved for human medicine are administered occasionally at the discretion of veterinarians. According to the 2021 JVARM report, 6.0 kg of carbapenems were distributed to small animal clinics, accounting for only 0.1% of the total quantity of antimicrobials approved for human medicine sold to such facilities [5]. The rarity of carbapenem-resistant isolates in this study may be explained by the antimicrobial use context in Japan. Although a small amount of human-approved carbapenems is occasionally used in companion animal clinics, carbapenem-class antimicrobials are not approved for veterinary use, resulting in minimal selection pressure for carbapenem resistance in this population. Given the concern over the selection of resistant strains due to the use of carbapenems, it is essential to refrain from using carbapenems in companion animals.

In this study, all three meropenem-resistant strains were non-*A. baumannii* species and carried carbapenemase genes on their chromosomes or plasmids. Two plasmids (pAC-2 and pAC-3_2) carrying carbapenemase genes were classified as the Rep_3 plasmid family. It has been reported that in *A. baumannii*, approximately one-quarter of Rep_3 family plasmids are associated with antimicrobial resistance genes, with carbapenemases being the most frequently detected [19].

In *A. radioresistens* AC-1, the only carbapenem resistance gene identified was the intrinsic gene *bla*_OXA-23_ located on the chromosome, and no other carbapenemase genes were detected. *bla*_OXA-23_ is an intrinsic resistance gene in *A. radioresistens*; however, it is known to be susceptible to carbapenems due to the absence of upstream insertion sequence *ISAba1* and usually low expression levels of the gene [20]. In *A. baumannii*, it has been reported that the insertion sequences such as *ISAba1* upstream of *bla*_OXA-51_, an intrinsic resistance gene that does not normally confer carbapenem resistance, can increase gene expression levels and lead to carbapenem resistance [21]. This suggests that the presence of *ISAba1* upstream of *bla*_OXA-23_ in strain AC-1 may contribute to increased carbapenem resistance gene expression.

The *bla*_OXA-58_ gene in pAC-2 and pAC-3_2, was flanked by a full-length copy of IS*Aba3* (794 bp) and a truncated copy of IS*Aba3* (427 bp). It has been reported that the expression of *bla*_OXA-58_ in *Acinetobacter* spp. is facilitated by promoter sequences containing insertion sequence elements, such as IS*Aba3* or its derivatives [22,23]. This study also suggested that *bla*_OXA-58_ expression was enhanced by the presence of IS*Aba3* upstream of the gene. Carbapenemases classified as Ambler’s class D, such as OXA-23 and OXA-58, have been reported to hydrolyze carbapenems but exhibit limited activity against third-generation cephalosporins [24,25,26]. In the present study, the AC-1 and AC-2 isolates, which harbored only class D carbapenemase genes, were also susceptible to cefotaxime.

In pAC-3_2, *bla*_NDM-1_ was also detected 4907 bp downstream of *bla*_OXA-58_, arranged in the structure *ISAba14-ble-bla_NDM-1_-ISAba125-aph(3′)-VI-ISAba14*. In a previous study in China, a similar *bla*_NDM-1_ containing structure was detected in a plasmid from *A. johnsonii* isolated from a human patient; however, the structure was oriented in the opposite direction and lacked one copy of *ISAba14* adjacent to the *ble* gene [27]. In addition, *bla*_NDM-1_ is located within the Tn125 composite transposon, which contains two *ISAba125* elements. Tn125 is known to be a major mechanism for the spread of *bla*_NDM_ genes in *Acinetobacter* species [28]. However, in this structure, only one *ISAba125* element was identified, and Tn125 was not detected. Nevertheless, the structure, flanked by *ISAba14* with inverted repeats, is likely to form a composite transposon. The absence of mobilization genes, such as MOBP and MOBQ, suggests that the mobility of plasmid pAC-3_2 itself is low. However, the composite transposon mediated by *ISAba14* may have mobility, potentially facilitating the transmission of resistance genes. Further investigation is needed to determine whether the composite transposons detected in this study exhibit actual mobility.

This study revealed the antimicrobial resistance profiles and genetic characteristics of carbapenem-resistant *Acinetobacter* spp. isolated from dogs and cats in Japan. However, since this study was conducted under the condition that all isolates were anonymized, detailed background information on the sampled animals, such as medical history and prior antimicrobial treatments was unavailable, which limited the ability to clarify the transmission routes of antimicrobial resistance genes. Carbapenem-resistant strains were detected at low frequencies and carried Ambler class B and D carbapenemase genes, which are likely expressed under the influence of adjacent insertion sequences. Notably, this is the first report of *bla*_NDM-1_- harboring *Acinetobacter* spp. isolated from companion animals in Japan. Although the detected carbapenem-resistant strains did not belong to *A. baumannii* which has a significant impact on human healthcare, there is concern that carbapenem resistance genes could be disseminated among *Acinetobacter* species or across different bacterial species via mobile genetic elements such as plasmids and insertion sequences.

This study has several limitations. Although the JVARM surveillance framework aimed to collect *Acinetobacter* spp. evenly from clinics across Japan, the final distribution of isolates was skewed toward submissions from urban regions, which may limit the geographic representativeness of the dataset. Detailed clinical information, including medical history, prior antimicrobial exposure, sex, and breed, was unavailable for most animals because all samples were anonymized as part of the surveillance framework. Only limited metadata (animal age and the fact that the isolates originated from different veterinary clinics) were available for the three meropenem-resistant isolates. These constraints limited our ability to investigate potential epidemiological links or to assess whether any isolates were associated with nosocomial infections. In addition, conclusions regarding the mobility of resistance gene–associated structures remain hypothetical because experimental validation (e.g., conjugation or transformation assays) was not performed.

These findings underscore the need to prevent the spread of antimicrobial resistance genes within companion animals and between animals and humans. In companion animal clinical settings, it is essential to implement appropriate hygiene practices, avoid the use of antimicrobials not approved for veterinary use, and promote the prudent use of antimicrobials. In addition, continuous surveillance and molecular characterization of resistance genes should be maintained. These efforts are critical for preventing the emergence and spread of antimicrobial resistance between animals and humans.

## 4. Materials and Methods

### 4.1. Sampling

As part of the Japanese Veterinary Antimicrobial Resistance Monitoring (JVARM) program, this study was conducted as a surveillance study. *Acinetobacter* spp. were collected from urine or skin samples obtained from diseased dogs and cats that were submitted for routine clinical diagnosis to four collaborating clinical laboratories from 133 veterinary clinics across Japan. A total of 139 isolates were collected, comprising 84 isolates from dogs and 55 from cats. All clinical samples were collected aseptically by veterinarians following standard procedures for urine collection and skin swabbing. To avoid duplicate sampling and to ensure that each isolate represented an independent clinical case, only one sample per animal per veterinary clinic was included. According to the JVARM framework for *Acinetobacter* spp., sampling is scheduled every three years, and if fewer than 30 strains per host are collected in a given year, additional sampling in the following year is conducted to reach the minimum required number for national surveillance analysis. Consistent with this protocol, sampling was conducted in 2020; however, because the target number of isolates was not reached in that year, additional sampling was carried out in 2021 to obtain sufficient numbers for surveillance analysis.

All isolates were anonymized before submission to our laboratory and used solely for monitoring and research purposes.

### 4.2. Informed Consent for Sampling

Clinical samples submitted to the clinical laboratories were sourced from veterinarians and owners and used under the agreement for research. All isolates were anonymized.

### 4.3. Identification

The samples were cultured on 5% sheep blood agar, chocolate agar, MacConkey agar, or BTB lactose agar. Bacterial identification was performed using the API 20NE system (bioMérieux, Tokyo, Japan) [29], the MicroScan WalkAway Plus system (Beckman Coulter, Inc., Tokyo Japan) [30], or MALDI-TOF MS (Bruker Daltonics, Bremen, Germany) [31] in each clinical laboratory and submitted to our laboratory. After that, those isolates were re-identified by MALDI-TOF MS using the Bruker Biotyper system with the Bruker Biotyper Reference Library (version 13; Bruker Daltonics). Species-level identification was assigned using the manufacturer’s recommended score threshold of ≥2.0. For MALDI-TOF MS identification, two independent colonies from each isolate were analyzed, each measured once, corresponding to duplicate measurements according to routine validated workflows. Isolates were preserved in 10% skim milk [32].

### 4.4. Antimicrobial Susceptibility Testing

The *Acinetobacter* spp. isolates were assessed for minimum inhibitory concentrations (MICs) against cefotaxime, meropenem, gentamicin, tetracycline, colistin, ciprofloxacin, and trimethoprim/sulfamethoxazole. MICs were determined using a standardized broth microdilution method following the Clinical and Laboratory Standards Institute (CLSI) standard M07: Methods for Dilution Antimicrobial Susceptibility Tests for Bacteria That Grow Aerobically (12th Edition; CLSI, Wayne, PA, USA) [33] using the cation-adjusted Mueller–Hinton broth (Mueller–Hinton Broth “Eiken”; Eiken Chemical Co., Ltd., Tokyo, Japan) and “Dry Plate ‘Eiken’” bacterial antimicrobial susceptibility testing reagent (EIKEN Chemical Co., Ltd., Tokyo, Japan). Concentration ranges were as follows: cefotaxime (0.5–64 μg/mL), meropenem (0.5–16 μg/mL), gentamicin (2–64 μg/mL), tetracycline (2–64 μg/mL), colistin (0.5–16 μg/mL), ciprofloxacin (0.06–8 μg/mL), and trimethoprim/sulfamethoxazole (19/1–38/2 μg/mL). Breakpoints defined in the CLSI document M100: Performance Standards for Antimicrobial Susceptibility Testing (34th Edition; CLSI) were applied [34]. As antimicrobial susceptibility in this surveillance was interpreted using CLSI clinical breakpoints for humans, in accordance with the JVARM framework and the Japanese national AMR surveillance system for humans, resistance rates reported here are comparable with those observed in human surveillance programs. *E. coli* ATCC 25922 was used as the quality control strain for tetracycline and trimethoprim/sulfamethoxazole, and *Pseudomonas aeruginosa* ATCC 27853 was used for the other antimicrobials. MIC testing for the meropenem-resistant isolates was performed twice for confirmation, whereas testing for all other isolates was performed once.

### 4.5. Whole-Genome Sequence and Analysis

Isolates exhibiting a minimum inhibitory concentration (MIC) of ≥8 μg/mL for meropenem were subjected to whole-genome sequencing using both short-read (Illumina, San Diego, CA, USA) and long-read (Nanopore, Oxford, UK) sequencing. This MIC threshold corresponds to the CLSI M100 (2024) meropenem resistance breakpoint.

For short-read sequencing, genomic DNA was extracted using the DNeasy Blood & Tissue Kit (Qiagen, Valencia, CA, USA) according to the manufacturer’s protocol. Genomic DNA was extracted from fresh overnight cultures grown on appropriate agar plates. Sequencing libraries were prepared using the Nextera XT DNA Library Preparation Kit (Illumina, Inc., San Diego, CA, USA). Whole-genome sequencing was performed in paired-end mode (2 × 300 bp) on MiSeq platform (Illumina, Inc., San Diego, CA, USA). Quality control and trimming of MiSeq raw reads was conducted using fastp v0.23.4.

For long-read sequencing, genomic DNA was extracted using the Wizard^®^ HMW DNA Extraction Kit (Promega, Fitchburg, WI, USA). Sequencing libraries were prepared using the Rapid Barcoding Kit 24 V14 (SQK-RBK114.24; Nanopore, Oxford, UK), with six to seven samples processed per sequencing run, with two barcodes per sample. Sequencing was performed on a MinION MK1B device using an R10.4.1 (FLO-MIN114) flow cell. Raw data were output in FAST5 and converted to POD5, then base-called in dorado (v 7.1.4). Hybrid assembly of short and long reads was conducted using Hybracter (v 0.11.2) [35], with hybracter hybrid --auto, with all other parameters kept at default. Assembly quality assessment and filtering incorporated contig length, coverage, and standard QUAST statistics [36]. Plasmid assembly and analysis were performed using Plassembler (v 1.6.2) [37]. Hybrid genome assembly was performed once per isolate based on established and widely adopted analysis pipelines. Assembly completeness and contamination were further evaluated using BUSCO (v5.8.3) [38] with the bacteria_odb10 lineage dataset and CheckM (v1.2.3) lineage workflow [39]. These tools were used to ensure that assembled genomes exhibited high completeness and low contamination prior to downstream genomic analyses.

Antimicrobial resistance genes were identified using AMRFinderPlus (v4.0.15) with the NCBI AMR gene database (database version 2024-12-18.1) [40]. Acquired resistance genes were called using the default AMRFinderPlus detection thresholds (≥90% identity and ≥80% coverage). Insertion sequences were identified using ISFinder [41]. Virulence factors and conjugative transfer elements, including the origin of transfer (*oriT*), type IV secretion system (T4SS), type IV coupling protein (T4CP) and genes encoding relaxase, were predicted using Bakta (v1.11.0) [42] with the full Bakta reference database release 6.0 (database date 24 February 2025). Plasmid prediction and reconstruction were carried out using MOB-suite (v3.1.9.), specifically MOB-Recon (v3.1.9.) [43]. Plasmid typing based on rep genes encoding plasmid replication initiation proteins was performed using Acinetobacter plasmid typing scheme (v2.0) [44]. Rep genes were detected by BLAST+ v2.13.0 using thresholds of ≥90% identity and ≥80% coverage to determine identity with known plasmids [45]. SnapGene Viewer software (v8.0.2) was used to visualize and manipulate sequences [46]. Figures were drawn to scale using the BLAST Ring Image Generator (BRIG) (v0.95) [47].

### 4.6. Statistical Analysis

Differences in antimicrobial resistance rates between dogs and cats were evaluated using Fisher’s exact test. A *p*-value of <0.05 was considered statistically significant.

### 4.7. Data Availability

Sequencing data are available in the DDBJ database under BioProject number PRJDB38022. Individual strain accession numbers are listed in Table S6.

## Figures and Tables

**Table 1:** Species distribution of *Acinetobacter* spp. isolates from diseased dogs and cats in Japan.

Species	Number of Isolates (%)		
	Dogs (*n* = 84)	Cats (*n* = 55)	Total (*n* = 139)
*A. baumannii*	19 (22.6)	16 (29.1)	35 (25.2)
*A. pittii*	18 (21.4)	11 (20.0)	29 (20.9)
*A. radioresistens*	12 (14.3)	4 (7.3)	16 (11.5)
*A. johnsonii*	5 (6.0)	5 (9.1)	10 (7.2)
*A. ursingii*	4 (4.8)	4 (7.3)	8 (5.8)
*A. lwoffii*	6 (7.1)	2 (3.6)	8 (5.8)
*A. junii*	3 (3.6)	4 (7.3)	7 (5.0)
*A. guillouiae*	2 (2.4)	3 (5.5)	5 (3.6)
*A. nosocomialis*	4 (4.8)		4 (2.9)
*A. calcoaceticus*	3 (3.6)		3 (2.2)
*A. bereziniae*	1 (1.2)	1 (1.8)	2 (1.4)
*A. haemolyticus*	2 (2.4)		2 (1.4)
*A. lactucae*	1 (1.2)	1 (1.8)	2 (1.4)
*A. schindleri*	1 (1.2)	1 (1.8)	2 (1.4)
*A. baylyi*	1 (1.2)		1 (0.7)
*A. dispersus*		1 (1.8)	1 (0.7)
*A. piscicola*	1 (1.2)		1 (0.7)
*A. proteolyticus*		1 (1.8)	1 (0.7)
*A. courvalinii*		1 (1.8)	1 (0.7)
*A. soli*	1 (1.2)		1 (0.7)
Sample type			
Urine	40 (47.6)	40 (72.7)	80 (57.6)
Skin	44 (52.4)	15 (27.3)	59 (42.4)

**Table 2:** MICs for *Acinetobacter* spp. isolated from diseased dogs and cats in Japan. To compare resistance rates between dogs and cats, *p*-values were determined by Fisher’s exact test. (* *p* < 0.05).

Antimicrobial Agents			Dogs (*n* = 84)			Cats (*n* = 55)		
	Range (µg/mL)	Breakpoint (µg/mL)	MIC50 (µg/mL)	MIC90 (µg/mL)	Number of Resistant Isolates (%)	MIC50 (µg/mL)	MIC90 (µg/mL)	Number of Resistant Isolates (%)
Meropenem	≤0.5–>16	8	≤0.5	1	1 (1.2%)	≤0.5	1	2 (3.6%)
Cefotaxime	≤0.5–>64	64	16	32	6 (7.1%)	16	>64	6 (10.9%)
Gentamicin	≤2–>64	16	≤2	2	6 (7.1%) *	≤2	32	12 (21.6%) *
Tetracycline	≤2–>64	16	≤2	4	6 (7.1%) *	4	64	13 (23.6%) *
Colistin	≤0.5–>16	4	≤0.5	1	0 (0.0%)	≤0.5	1	1 (1.8%)
Ciprofloxacin	≤0.06–>8	4	0.25	>8	13 (15.5%) *	0.5	>8	20 (36.4%) *
Sulfamethoxazole/ Trimethoprim	≤9.5/0.5–>152/8	76/4	≤9.5/0.5	152/8	13 (15.5%) *	≤9.5/0.5	>152/8	18 (32.7%) *

**Table 3:** Isolation information and molecular characterization of the genome of carbapenem-resistant *Acinetobacter* spp. derived from dogs and cats in Japan. Only plasmids carrying antibiotic resistance genes are shown.

Strains						
AC-1	Species	*A. radioresistens*				
	Year	2023				
	Origins	Dog				
	Sampling sites	Urine				
	Meropenem MIC (µg/mL)	8				
	Genome	Location	Replicon Type	Size (bp)	Genes	Antimicrobial agent class
		Chromosome	ND	3,253,141	*bla*_OXA-23_	Carbapenems
		pAC-1	ND	6078	*ant(2″)-Ia*	Aminoglycosides
AC-2	Species	*A. proteolyticus*				
	Year	2021				
	Origins	Cat				
	Sampling sites	Urine				
	Meropenem MIC (µg/mL)	16				
	Genome	Location	Replicon Type	Size (bp)	Genes	Antimicrobial agent class
		Chromosome	ND	4,580,074	*bla*_OXA-674_	Carbapenems
					*aac(6′)-I*	Aminoglycosides
		pAC-2	r3-T61	10,429	*bla*_OXA-58_	Carbapenems
AC-3	Species	*A. johnsonii*				
	Year	2023				
	Origins	Cat				
	Sampling sites	Urine				
	Meropenem MIC (µg/mL)	>16				
	Genome	Location	Replicon Type	Size (bp)	Genes	Antimicrobial agent class
		Chromosome	ND	3,288,835	*bla*_OXA-651_	Carbapenems
		pAC-3_1	ND	370,800	*tet(H)*	Tetracyclines
		pAC-3_2	r3-T20	54,777	*bla*_OXA-58_	Carbapenems
					*bla*_NDM-1_	Carbapenems
					*dfrA1*	Trimethoprim
					*aac(3)-IId*	Aminoglycosides
					*aph(3′)-VI*	Aminoglycosides
		pAC-3_3	ND	4135	*aadA27*	Aminoglycosides

## Data Availability

The whole-genome sequencing data generated in this study are available in the DNA Data Bank of Japan (DDBJ) under BioProject accession number PRJDB38022. Individual strain accession numbers are provided in Table S6.

## Supplementary Materials

The following supporting information can be downloaded at: https://www.mdpi.com/article/10.3390/antibiotics15040329/s1, Table S1: MIC distributions (counts per two-fold dilution step) for seven antimicrobials among *Acinetobacter* isolates; Table S2: Antimicrobial resistance rates by sampling origin (urine vs. skin) in dogs and cats; Table S3: Sequencing read metrics for three meropenem-resistant *Acinetobacter* isolates; Table S4: Hybrid genome assembly quality metrics; Table S5: Per-replicon sequencing depth for short and long reads; Table S6: Summary of sequencing data and accession numbers.
