# Supplementary material for: Genetic Characterization of Carbapenem-Resistant Acinetobacter spp. Isolated from Diseased Companion Animals in Japan
Source: Antibiotics (Basel). 2026 Mar 24;15(4):329. doi: 10.3390/antibiotics15040329 (PMC13113104; doi:10.3390/antibiotics15040329)
Supplement: Supplementary file 1 [file antibiotics-15-00329-s001.zip › Table S3.pdf]

Table S3. Sequencing read metrics for three meropenem-resistant *Acinetobacter* isolates

	AC-1	AC-2	AC-3
Miseq_DNA concentration (ng/μL)	18.2	11.8	29.1
Miseq_RawSeq_Mean_Quality (Q)	36.02	35.35	36.1
Miseq_RawSeq_Median_Quality (Q)	37.3	36.32	37.33
Miseq_RawSeq_Mean_Length (bp)	212.40	280.88	208.18
Miseq_RawSeq_Total_Reads	4,371,744	1,165,930	4,077,388
Miseq_RawSeq_Total_Bases (Mb)	928.55	327.49	848.85
Miseq_RawSeq_GC_Content (%)	43.13	41.73	42.3
MiNION_DNA concentration (ng/μL)	42.8	91.4	56.0
MinION_RawSeq_Mean_Quality (Q)	18.86	18.31	18.82
MinION_RawSeq_Median_Quality (Q)	19.84	15.56	20.01
MinION_RawSeq_Mean_Length (bp)	4216.21	4817.86	5817.83
MinION_RawSeq_N50 (bp)	8,683	28,819	13,802
MinION_RawSeq_Total_Reads	115682	10265	39051
MinION_RawSeq_Total_Bases (Mb)	487.74	49.46	227.19
MinION_RawSeq_GC_Content (%)	42.19	41.59	41.22
