# Supplementary material for: Genetic Characterization of Carbapenem-Resistant Acinetobacter spp. Isolated from Diseased Companion Animals in Japan
Source: Antibiotics (Basel). 2026 Mar 24;15(4):329. doi: 10.3390/antibiotics15040329 (PMC13113104; doi:10.3390/antibiotics15040329)
Supplement: Supplementary file 1 [file antibiotics-15-00329-s001.zip › Table S4.pdf]

Table S4. Hybrid genome assembly quality metrics

	AC-1	AC-2	AC-3
Hybrid_Assembly_QUAST_total_length (bp)	3,344,824	4,967,702	3,745,199
Hybrid_Assembly_QUAST_N50 (bp)	3,253,141	2,506,408	3,288,904
Hybrid_Assembly_QUAST_L50	1	1	1
Hybrid_Assembly_QUAST_largest_contig (bp)	3,253,141	2,506,408	3,288,904
Hybrid_Assembly_QUAST_contigs	8	11	5
Hybrid_Assembly_QUAST_GC (%)	41.7	40.89	41.07
Hybrid_Assembly_CheckM_completeness (%)	100	99.8630137	100
Hybrid_Assembly_CheckM_contamination (%)	0.404435747	0.589159699	0.906718852
Hybrid_Assembly_BUSCO_complete (%)	100	99.2	100
Hybrid_Assembly_BUSCO_missing (%)	0	0	0
