# Supplementary material for: Genetic Characterization of Carbapenem-Resistant Acinetobacter spp. Isolated from Diseased Companion Animals in Japan
Source: Antibiotics (Basel). 2026 Mar 24;15(4):329. doi: 10.3390/antibiotics15040329 (PMC13113104; doi:10.3390/antibiotics15040329)
Supplement: Supplementary file 1 [file antibiotics-15-00329-s001.zip › Table S5.pdf]

Table S5. Per-replicon sequencing depth for short and long reads

	AC-1		AC-2		AC-3			
contig	chromosome	pAC-1	chromosome	pAC-2	Chromosome	pAC-3_1	pAC-3_2	pAC-3_3
length (bp)	3,253,136	6,078	4,578,799	10,429	3,288,843	37,0800	54,777	4,135
mean_depth_short	246.09	1132.63	62.21	121.72	225.66	207.99	81.77	2201.84
sd_depth_short	86.71	430.84	20.68	54.84	61.99	63.82	32.11	443.97
q25_depth_short	197	726	52	83	195	170	60	1,958
q75_depth_short	306	1,500	75	162	265	252	102	2,508
mean_depth_long	82.98	0	58.22	715.22	56.12	74.91	153.85	1155.74
sd_depth_long	12.8	0	12.86	109.32	9.54	9.95	13.82	84.2
q25_depth_long	75	0	49	658	50	68	148	1,101
q75_depth_long	91	0	66	785	61	81	163	1,228
