# Supplementary material for: Genetic Characterization of Carbapenem-Resistant Acinetobacter spp. Isolated from Diseased Companion Animals in Japan
Source: Antibiotics (Basel). 2026 Mar 24;15(4):329. doi: 10.3390/antibiotics15040329 (PMC13113104; doi:10.3390/antibiotics15040329)
Supplement: Supplementary file 1 [file antibiotics-15-00329-s001.zip › Table S6.pdf]

Table S6. Summary of sequencing data and accession numbers

Strains	Sample Name	Species	Sequencing Platform	BioProject	BioSample	Run(DRR)
AC-1	R5-AC-DG-6	*A. radioresistens*	Illumina MiSeq	PRJDB38022	SAMD01708908	DRR893424
AC-1	R5-AC-DG-6	*A. radioresistens*	Oxford Nanopore	PRJDB38022	SAMD01708908	DRR893425
AC-2	R3-AC-F-9	*A. proteolyticus*	Illumina MiSeq	PRJDB38022	SAMD01708907	DRR893422
AC-2	R3-AC-F-9	*A. proteolyticus*	Oxford Nanopore	PRJDB38022	SAMD01708907	DRR893423
AC-3	R5-AC-F-16	*A. johnsonii*	Illumina MiSeq	PRJDB38022	SAMD01708909	DRR893426
AC-3	R5-AC-F-16	*A. johnsonii*	Oxford Nanopore	PRJDB38022	SAMD01708909	DRR893427
