# Supplementary material for: Genetic Characterization of Carbapenem-Resistant Acinetobacter spp. Isolated from Diseased Companion Animals in Japan
Source: Antibiotics (Basel). 2026 Mar 24;15(4):329. doi: 10.3390/antibiotics15040329 (PMC13113104; doi:10.3390/antibiotics15040329)
Supplement: Supplementary file 1 [file antibiotics-15-00329-s001.zip › Table S1.pdf]

Table S1. MIC distributions (counts per two-fold dilution step) for seven antimicrobials among *Acinetobacter* isolates

All isolates combined（n=139）

Antimicrobial agents	Range (μg/mL)	Breakpoint (μg/mL)	Number of Isolates(n)											
			MIC （μg/mL)											
			≤0.06	0.125	0.25	0.5	1	2	4	8	16	32	64	>64
Meropenem	≤0.5->16	8				108	24	4	0	1	1	1		
Cefotaxime	≤0.5->64	64				1	4	9	13	35	43	22	4	8
Gentamicin	≤2->64	16						116	5	0	8	2	1	7
Tetracycline	≤2->64	16						85	32	3	3	6	2	8
Colistin	≤0.5->16	4				107	24	7	1	0	0	0		
Ciprofloxacin	≤0.06->8	4	9	29	32	30	4	2	1	4	28			

Antimicrobial agents	Range (μg/mL)	Breakpoint (μg/mL)						
Sulfamethoxazole /Trimethoprim	≤9.5/0.5 - >152/8	76/4	≤9.5/0.5	19/1	38/2	76/4	152/8	>152/8
			99	5	5	5	8	17

Dogs only (n=84)

Antimicrobial agents	Range (μg/mL)	Breakpoint (μg/mL)	Number of Isolates(n)											
			MIC （μg/mL)											
			≤0.06	0.125	0.25	0.5	1	2	4	8	16	32	64	>64
Meropenem	≤0.5->16	8				66	14	3	0	1	0	0		
Cefotaxime	≤0.5->64	64				0	4	6	8	23	26	11	4	2
Gentamicin	≤2->64	16						77	1	0	3	0	0	3
Tetracycline	≤2->64	16						61	15	2	1	1	1	3
Colistin	≤0.5->16	4				66	14	4	0	0	0	0		
Ciprofloxacin	≤0.06->8	4	8	23	18	19	3	0	0	1	12			

Antimicrobial agents	Range (μg/mL)	Breakpoint (μg/mL)						
Sulfamethoxazole /Trimethoprim	≤9.5/0.5 - >152/8	76/4	≤9.5/0.5	19/1	38/2	76/4	152/8	>152/8
			66	3	2	2	4	7

Cats only (n=55)

Antimicrobial agents	Range (μg/mL)	Breakpoint (μg/mL)	Number of Isolates(n) MIC （μg/mL)											
			≤0.06	0.125	0.25	0.5	1	2	4	8	16	32	64	>64
Meropenem	≤0.5->16	8				42	10	1	0	0	1	1		
Cefotaxime	≤0.5->64	64				1	0	3	5	12	17	11	0	6
Gentamicin	≤2->64	16						39	4	0	5	2	1	4
Tetracycline	≤2->64	16						24	17	1	2	5	1	5
Colistin	≤0.5->16	4				41	10	3	1	0	0	0		
Ciprofloxacin	≤0.06->8	4	1	6	14	11	1	2	1	3	16			
Sulfamethoxazole /Trimethoprim	≤9.5/0.5 - >152/8	76/4	≤9.5/0.5	19/1	38/2	76/4	152/8	>152/8						
			33	2	3	3	4	10						
