# Supplementary material for: Genetic Characterization of Carbapenem-Resistant Acinetobacter spp. Isolated from Diseased Companion Animals in Japan
Source: Antibiotics (Basel). 2026 Mar 24;15(4):329. doi: 10.3390/antibiotics15040329 (PMC13113104; doi:10.3390/antibiotics15040329)
Supplement: Supplementary file 1 [file antibiotics-15-00329-s001.zip › Table S2.pdf]

Table S2. Antimicrobial resistance rates by sampling origin (urine vs skin) in dogs and cats. To compare resistance rates between urine and skin, p-values were determined by Fisher's exact test. (*p < 0.05)

Antimicrobial agents	Range (μg/mL)	Breakpoint (μg/mL)	Number of resistant isolates (%)					
			Dogs (n=84)		Cats (n=55)		Total (n=139)	
			Urine (n=40)	Skin (n=44)	Urine (n=40)	Skin (n=15)	Urine (n=80)	Skin (n=59)
Meropenem	≤0.5->16	8	1 (2.5%)	0 (0.0%)	2 (5.0%)	0 (0.0%)	3 (3.8%)	0 (0.0%)
Cefotaxime	≤0.5->64	64	5 (12.5%)	1 (2.3%)	5 (12.5%)	1 (6.7%)	10 (12.5%)	2 (3.4%)
Gentamicin	≤2->64	16	5 (12.5%)	1 (2.3%)	9 (22.5%)	3 (20.0%)	14 (17.5%)	4 (6.8%)
Tetracycline	≤2->64	16	2 (5.0%)	4 (9.1%)	11 (27.5%)	2 (13.3%)	13 (16.3%)	6 (10.2%)
Colistin	≤0.5->16	4	0 (0.0%)	0 (0.0%)	1 (2.5%)	0 (0.0%)	1 (1.3%)	0 (0.0%)
Ciprofloxacin	≤0.06->8	4	9 (22.5%)	4 (9.1%)	16 (40.0%)	4 (26.7%)	25 (31.3%)*	8 (13.6%)*
Sulfamethoxazole /Trimethoprim	≤9.5/0.5 - >152/8	76/4	6 (15.0%)	7 (15.9%)	15 (37.5%)	3 (20.0%)	21 (26.3%)	10 (16.9%)
